# pNiPAM-Nanoparticle-Based Antiapoptotic Approach for Pro-Regenerative Capacity of Skeletal Myogenic Cells

**DOI:** 10.3390/nano11102495

**Published:** 2021-09-24

**Authors:** Magdalena Nowaczyk, Agnieszka Zimna, Tobiasz Deptuła, Katarzyna Fiedorowicz, Natalia Rozwadowska, Marta Podralska, Maciej Kurpisz

**Affiliations:** Institute of Human Genetics, Polish Academy of Sciences, 60-479 Poznan, Poland; magdalena.przybyl@igcz.poznan.pl (M.N.); agnieszka.zimna@igcz.poznan.pl (A.Z.); tobia0@gmail.com (T.D.); katarzyna.fiedorowicz@igcz.poznan.pl (K.F.); natalia.rozwadowska@igcz.poznan.pl (N.R.); marta.podralska@igcz.poznan.pl (M.P.)

**Keywords:** nanoparticles, pNiPAM, apoptosis, necrosis, bax channel blocker, skeletal muscle derived stem/progenitor cells

## Abstract

The biocompatibility of pNiPAM (Poly N-isopropylacrylamide) copolymers has been examined and they did not exert any cytotoxic effects. Their properties and vulnerable temperature characteristics make them candidates for use in medical applications. We synthesized a well-characterized nanoparticles-based cargo system that would effectively deliver a biological agent to human skeletal myogenic cells (SkMCs); among other aspects, a downregulating apoptotic pathway potentially responsible for poor regeneration of myocardium. We confirmed the size of the pNiPAM based spheres at around 100 nm and the nanomeric shape of nanoparticles (NP) obtained. We confirmed that 33 °C is the adequate temperature for phase transition. We performed the dynamics of cargo release. A small amount of examined protein was detected at 10 min after reaching LCTS (lower critical solution temperature). The presented results of the test with BSA (bovine serum albumin) and doxorubicin loaded into nanoparticles showed a similar release profile for both substances. SkMCs incubated with NP loaded with antiapoptotic agent, BCB (Bax channel blocker), significantly diminished cell apoptosis (*p* < 0.01). Moreover, the lowest apoptotic level was detected in SkMCs treated with camptothecin and simultaneously incubated with pNiPAMs loaded with BCB. Application of nanoparticles loaded with BCB or subjected to BCB alone did not, however, diminish the amount of apparently necrotic cells.

## 1. Introduction

An increased interest in nanotechnology in general and rapid development of nanoparticle technology has led to progress in various research fields in the past decade. The interest in nanoparticles was due to great potential to be applied in novel approaches to clinical diagnosis and treatment. Their unique physical, optical, and electronic features may provide high activity and unusual properties through nanotechnology means [1]. It seems clear that in recent years nanoparticles are popular not only with respect to their potential bioapplications (i.e., drug delivery, biosensors, and bioscaffolds) but also due to the intense development of nanomaterials themselves resulting in numerous model systems as the probes to investigate and/or detect different physical phenomena [2,3,4]. None of the less polymeric nanoparticles have been widely used for biological applications including drug delivery since they must be responsive to external stimuli such as temperature and pH [5]. Such behavior is particularly preferred for the targeted delivery of drugs and active biological substances. Among many polymeric structures, pNiPAM nanoparticles are well-known and well-characterized. The biocompatibility of pNiPAM copolymers has been widely studied, although cytotoxicity of the monomer is questionable. It is clear that the extent to which it is toxic to cells at the concentration of 5 mg/mL in the culture media depends on the cell type [6]. However, in the previous study by Deptuła et al., they showed that pNiPAM nanoparticles do not exhibit any cytotoxic effects on the investigated cell lines and that nanoparticle-based scaffolds promoted cell growth [7]. pNiPAM copolymers exhibit low critical solution temperature above which polymer–solvent interactions change from hydrophilic to hydrophobic. The interstitial spaces in hydrogel particles are filled with water and the nanoparticles stay swollen, above the transition temperature polymer–polymer interaction and the polymeric particles start to collapse releasing the water trapped inside resulting in small nanospheres. This temperature-dependent process is fully reversible; as the temperature returns below the LCST (lower critical solution temperature), the interactions between polymer and solvent improve and nanoparticles swell again. Additionally, the fact that the transition temperature of pNiPAM based copolymers is very close to the human body temperature makes them a good candidate for use in medical applications.

Cardiovascular diseases (CVD) are the main causes of mortality worldwide. Myocardial infarction (MI) is mainly caused by the blockage of coronary arteries and results in a loss of cardiac cells, which subsequently leads to the formation of a postinfarction scar. These structural changes result in a process called ventricular remodeling in which one can observe thinning of the ventricular wall, which could eventually result in cardiac failure. Classical heart failure (HF) therapy is still mainly based on surgical interventions and complementary pharmacological treatments, which never fully restore the physiological function of the myocardium. Scientific evidence demonstrates that apoptotic cell death plays a key role in the development of heart failure [8,9]. Apoptosis is a biological process that develops naturally and is regulated by the prodeath and prosurvival balance in living cells. This highly regulated and complex process has been reported to play a crucial role in heart treatment. Thus, the apoptotic process of applied stem/progenitor cell grafting has been considered as a process limiting myocardium regeneration. There have been countless papers covering in detail the mechanisms of apoptosis, and it can be described in general as a process leading to activation of a caspases cascade, which may finally execute the process, with two major apoptotic pathways, the ‘intrinsic’ one, which utilizes mitochondria and the ‘extrinsic’, which utilizes membrane receptors (i.e., FAS and FADD). Both ways, transducing apoptotic signaling results in cell degradation. In this study, we focused on the intrinsic (mitochondria) pathway mediated by the Bcl-2 proteins family in particular. This set of proteins consists of both prosurvival and prodeath factors. Since apoptosis is a key component in HF, myocyte survival and a possible antiapoptotic mechanism mediated through nanotechnological means are at the center of attention.

The apoptosis regulator Bax (Bcl-2-like protein 4) is a member of the Bcl-2 gene family. Bax is believed to interact with and induce the opening of the mitochondrial voltage-dependent anion channel (VDAC) [10]. Alternatively, growing evidence also suggests that activated Bax and/or Bak form oligomeric pores, a mitochondrial apoptosis-induced channel in the mitochondrial outer membrane. This results in the release of cytochrome c and other proapoptotic factors from the mitochondria [11] often referred to as mitochondrial outer membrane permeabilization, leading to activation of caspases [12]. Bax activation is stimulated by various abiotic factors, including heat, hydrogen peroxide, low or high pH, and mitochondrial membrane remodeling. In addition, it can become activated antiapoptotically by Bcl-2 sequestrate. To induce that pathway, we used a Bax channel blocker (BCB) trapped as a carrier within pNiPAM-HEMA-AA-oligoLA nanoparticles.

The aim of this study included: I) to synthesize a well-characterized nanoparticle-based drug system that would effectively deliver a biological agent to human skeletal myogenic cells; II) to downregulate the apoptosis pathway responsible for poor regeneration of myocardium and/or grafted cells. The proregenerative ability of skeletal myogenic cells (SkMCs) in a failing heart would be an essential part of the restoration of myocardial physiological function. The regenerative process, which is naturally occurring in every tissue, including the heart, has been diminished through activation of programmed cell death (apoptosis). In delivered cellular graft and recipient tissues, we believe, that apoptosis suppression performed in a controlled way may limit the loss of both cardiac myoblasts and implemented SkMCs associated with an intervention in a failing heart.

## 2. Materials and Methods

### 2.1. Synthesis of pNiPAM-HEMA-AA-oligoLA

All chemicals were purchased from Sigma-Aldrich (Saint Louis, MO, USA) unless otherwise stated. The hydrogel was synthesized from N-isopropylacrylamide (NiPAM), a (2-hydroxylethylmethacrylate) HEMA, and oligolactide (AA-oligoLA). pNiPAM-HEMA-AA-OligoLA copolymer was synthesized by free radical polymerization. Monomers, NIPAAm (6 g, 0.053 mol), HEMA, and oligoLA, with a certain molar ratio (84/10/6) were dissolved in 50 mL of 1, 4-dioxane. The polymerization was carried out at 70 °C for 24 h in an argon atmosphere. The copolymer was precipitated in hexane and further purified by precipitation from tetrahydrofuran (THF) into diethyl ether and dried. The macromer was synthesized by a two-step method. Firstly, NaOCH_3_ as an initiator was used for generating OligoLA by ring-opening polymerization of lactide. A total of 25 g of D, L-lactide (0.193 mol) was dissolved in 50 mL of CH_2_Cl_2_ and put into the glass flask. One gram of NaOCH_3_ (0.019 mol) dissolved in methanol was then added. The reaction was performed at 0 °C for 2 h. The solution was neutralized with 0.1 M HCL and washed with DI (deionized) water. Then, we evaporated the organic layer at 60 °C, and the oligoLA was obtained. The average number of LA units in each oligomer was confirmed by 1H NMR (Nuclear Magnetic Resonance) (around 3). In the second step, oligoLA was esterified using acryloyl chloride. A total of 32.4 g of oligoLA (151.3 mmol) was dissolved in 50 mL of CH_2_Cl_2_. Then we added 23.6 mL of triethylamine triethylamine (169 mmol). Acryloyl chloride (13.6 mL, 169 mol) was added dropwise for 1 h until the mixture cooled down. The mixture was stirred overnight at room temperature, and then rinsed with 0.2 M Na_2_CO_3_, 0.1 M HCl, and DI water respectively. The AA-oligoLA was obtained by evaporating the solvent at 40 °C and washed using an ethyl acetate/CH_2_Cl_2_ mixture. Poly (NiPAM-co-HEMA-co-AA-oligoLA) was synthesized by free radical polymerization by the following procedure. Stoichiometric amounts of NIPAAm, HEMA, and AAoligoLA (molar ratio, respectively, 86/10/6) were dissolved in 100 mL of dioxane in a 250 mL three-necked flask. Then, the initiator benzoyl peroxide was added. The polymerization was performed at 60 °C overnight. The hexane was used for solution precipitation. The polymer was purified twice using THF/ethyl ether.

Two-step synthesis of Poly(NiPAM-co-HEMA-co-AA-oligoLA).
  a) Generation of OligoLactide by ring-opening polymerization:
    - Dissolving of 50 g D, L- lactide in 100 mL CH_2_Cl_2_ (one-necked flask);
    - Addition of 1 g NaOCH_3_ dissolved in methanol (intense stirring);
    - 0 °C for 2 h;
    - Neutralization by rinsing with 0.1M HCl and washing with deionized water;
    - Evaporation of the organic layer at 60 °C (rotary evaporation).
  b) Estrification of OligoLactide:
    - Dissolving of OligoLA in 100 mL CH_2_Cl_2_;
    - Addition of Trimethylamine;
    - Cooling to 0 °C;
    - Addition of Acryl chloride dropwise (1h);
    - Stirring overnight at 20 °C;
    - Rinsing with 0.2M NA_2_CO_3,_ HCl and deionized water;
    - Evaporation of the solvent at 40 °C;
    - Purification using chromatography with ethyl acetate/CH_2_Cl_2_ as eluent.
  c) Free radical polymerization:
    - Dissolving of NiPAM, HEMA, AA-oligoLA (86/10/4) in 100 mL of dioxane (three-necked flask);
    - Addition of Benzoyl peroxide as an initiator;
    - Stirring at 60 °C for 8–12 h;
    - Precipitation in hexane;
    - Purification twice with THF/ethyl ether.

### 2.2. SEM—Scanning Electron Microscopy

The morphology of the synthesized nanoparticles was assessed by using a scanning electron microscopy, performing SEM measurements. For scanning electron microscopy imaging of the micrographs, the accelerating voltage 15 kV and secondary electron (SEI) mode was used (Cryo-SEM, Jeol, JSM 7001F TTLS, Richland, WA, USA). Samples of a low concentration (1 mg of stock solution in 1 mL of water) were deposited on a glass plate and coated with platinum using a sputtering system (Quorum Technologies PP3000T, East Sussex, UK) for 60 s to provide an electrically conductive thin film to reduce thermal damage and charge the samples. The platinum-coated nanoparticles were then vacuum-dried and examined. 

### 2.3. Light Scattering

As a result of the light scattering experiment, a water-soluble nanoparticle with nanometric size was determined. The hydrodynamic radius of the polymeric nanoparticles was measured by dynamic light scattering (DLS) using the green laser at a wavelength of 532 nm as the light source in a dilute regime (nanomoles) in water. The photon correlation was performed by an ALV-5000/E photon correlator (ALV-Laser Vertriebsgesellschaft, GmbH, Langen, Germany). The scattering intensity was measured 5 times and each measurement itself took 200 s. 

### 2.4. Zeta Potential

Measuring the Zeta potential took place with NanoSight NTA (Malvern Panalytical, Malvern, UK). A customized chamber of low concentration (10−6) was fitted with platinum electrodes, which allowed a variable electric field to be applied to a sample. This caused particle motion and the Z-NTA recorded the apparent drift velocity for each particle. Performed observations of the total velocity at different depths within the sample chamber enable separation of components to obtain the Zeta potential.

### 2.5. Differential Scanning Calorimetry (DSC) Measurements

The nature of the previously synthesized copolymer was assessed by heat measurements carried out by means of DSC on a DSC8000 (PerkinElmer, Mundelein, IL, USA) calorimeter. The DSC runs were recorded while heating and cooling the samples at a temperature rate of 10 K/min. Dynamic differential scanning calorimetry was performed and low critical solution temperature data were provided. 

### 2.6. Load and Release Examination

#### 2.6.1. Bovine Serum Albumin (Model Protein) Load and Release from the Hydrogel

The synthesized hydrogel polymer was dissolved in water to prepare 10% *w*/*v* hydrogel solution. The change in temperature caused the NiPAM to change its properties from hydrophobic to hydrophilic and was responsible for the mechanism of swelling. Load and release examinations confirmed its swelling. The solution was then thoroughly mixed with BSA (Sigma-Aldrich, Saint Louis, MO, USA). The final concentrations of BSA were 6 mg/mL and 60 mg/mL, respectively. In order to release the water trapped inside the nanoparticles and replace it with BSA, the mixtures were first heated up to 38 °C, then cooled down to room temperature, and left overnight to achieve full swelling. The dispersed phase was separated from the continuous phase by means of centrifugation (17,500 rpm for 30 min). The supernatants were removed and replaced with water. Then the hydrogel mixture was heated up above LCST. At predetermined time points, the released supernatant was collected and the BSA concentration measured using prestained gradient gel for electrophoresis.

#### 2.6.2. Doxorubicin (Model Drug) Load and Release from the Hydrogel

Doxorubicin is an anthracycline, which inhibits topoisomerase II to block DNA and RNA replication in mammalian cells. Therefore, it is used as an effective anticancer agent. It accumulates in the nucleus, intercalates with DNA, and acts as a cytostatic and proapoptotic agent in cancer cells. The production of free radicals and oxidative stress is highly involved in the toxic and anticancer mechanisms of Doxorubicin [13,14]. The clinical application of Doxorubicin has some limitations due to its serious side effects and development of resistance [15]. Thus, delivery of Doxorubicin (Sigma-Aldrich, Saint Louis, MO, USA) via nanoparticles may be an efficient way to overcome such limitations. Generally, intracellular localization of nanoparticles mostly occurs in the cytoplasm and less in nuclei. In fact, nuclear transport of nanoparticles is challenging since the nuclear pores have a specific diameter of 30−40 nm and the diffusion rate through such small pore size depends on the nanoparticle size [12]. The synthesized hydrogel polymer was dissolved in water to prepare 10% *w*/*v* hydrogel solution. Then the doxorubicin (1 mg/mL) was added and mixed. In order to release the water trapped inside of nanoparticles and replace it with doxorubicin solution, the mixture was first heated up to 38 °C, then cooled down to room temperature, and left overnight to achieve full swelling. The dispersed phase was separated from the continuous phase by means of centrifugation (17,500 rpm for 30 min). The supernatant was removed and replaced with water. Then the hydrogel mixture was heated up above LCST and after a defined period of time, the supernatant was collected and the released model drug was assayed employing GloMax^®^-Multi Fluorescence Module (Promega, Madison, WI, USA). The fluorescence was excited at 490 nm and intensity was detected at 510−570 nm filter. Leading and release data are presented in the respective tables (Results).

### 2.7. Skeletal Myogenic Cells (SkMCs) In Vitro Culture and Apoptosis/Necrosis Induction

SkMCs were obtained from the patient from remaining muscle tissue after a cruciate ligament reconstruction procedure. Skeletal myogenic cells were isolated and enriched using a previously described methodology called “pre-plating” with gelatin coated flasks [16]. All procedures here employed were approved by the Local Bioethical Committee of Poznan Medical University (permission no. 818/13) and were conducted in accordance with the principles of Good Clinical Practice. Written consents from the patients were obtained. All the methods used in the study with human biological material adhered to the principles outlined in the Declaration of Helsinki. Cells were cultured in standard Dulbecco’s modified Eagle’s medium with 4.5 g/L of glucose, supplemented with 20% bovine fetal serum (Lonza Group, Basel, Switzerland), 1% antibiotics, 1% Ultraglutamine, and bFGF (Sigma-Aldrich, St. Louis, MO, USA). Cells were maintained in vitro in standard cell culture conditions (95% humidity and 5% CO_2_ at 37 °C). The medium was changed every 48 h, and to avoid spontaneous myotube formation the cells were passaged when they reached 70% confluence using 0.25% trypsin (Lonza Group, Basel, Switzerland). Cells were in vitro cultured until they reached appropriate distortion quantity and confluence, then the polymeric agent was applied for 24 h, either loaded with BCB or without cargo.

### 2.8. Cell Apoptosis/Necrosis

The aim of the present study was to verify the antiapoptotic features of the pNiPAM polymer loaded with BCB (50 µM) (Calbiochem, San Diego, CA, USA), employing camptothecin (5 µM) (Thermo Fisher Scientific, Waltham, MA, USA) as an apoptosis inducer and a high temperature as a necrosis (and irreversible apoptosis) inducer. Due to its low cost and easy accessibility, this material has been used extensively in the pharmaceutical, chemical, light, and food industries for decades. During the experiment, in order to induce apoptosis, SkMCs were exposed to camptothecin (Thermo Fisher Scientific, Waltham, MA, USA) overnight and then examined by flow cytometry using Annexin V staining. Briefly, Annexin V binds surface phosphatidylserine. These phospholipids become exposed to the cell surface during early stages of apoptosis. Annexin V is linked to FITC (fluorescein) and labels apoptotic cells [17] while necrotic cells have been marked by 7-AAD. The analysis was performed using the Amnis FlowSight flow cytometer (Beckman Coulter, Brea, CA, USA).

## 3. Results

### 3.1. Scanning Electron Microscope Evaluation of Nanoparticles

**Figure 1:**
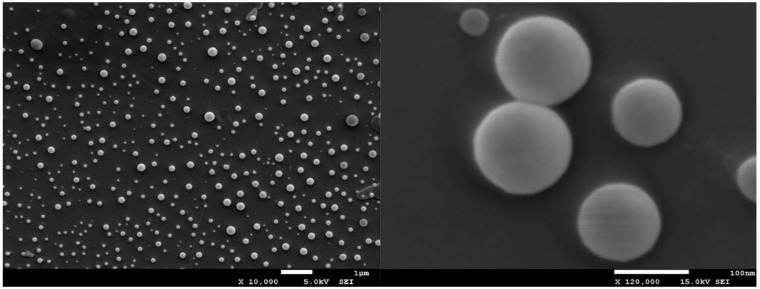
pNiPAM-HEMA-AA-oligoLA spherical nanoparticles’ evaluation in scanning electron microscopy.

According to SEM experiments, we confirmed that the size of the pNiPAM based spheres was around 100 nm, which was consistent with the light scattering experiment. Moreover, spherical shape and homogenous size distribution were determined (Figure 1).

### 3.2. Dynamic Light Scattering

**Figure 2:**
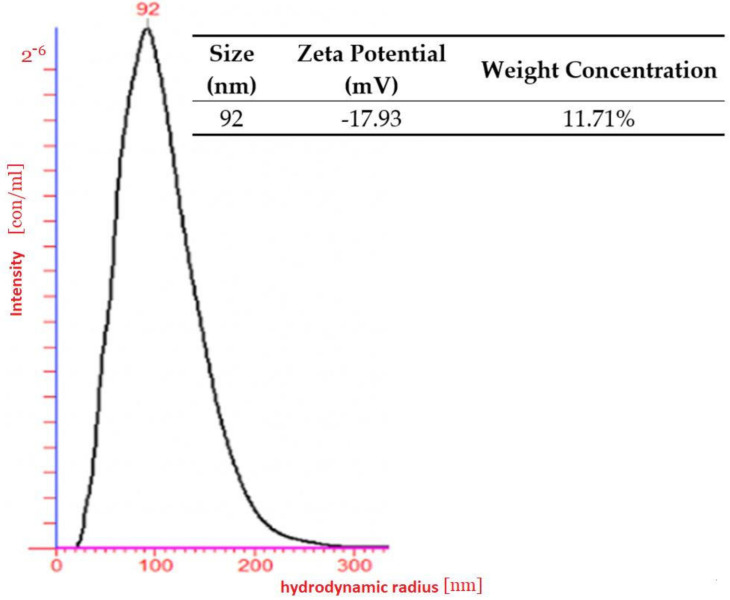
Hydrodynamic radius and size distribution of pNiPAM-HEMA-AA-oligoLA spherical nanoparticles.

The results obtained from DLS (dynamic light scattering) confirmed the nanometric shape of nanoparticles. The average size of the obtained particles was around 100 nm according to hydrodynamic analysis (Figure 2). The diameter was measured using Dynamic Light Scattering and was given as a hydrodynamic radius. The hydrodynamic radius of the polymeric nanoparticles in dilute regime (nanomoles) in water measured by DLS used the green laser at a wavelength of 532 nm as the light source in a dilute regime in water. The photon correlation was performed by an ALU-5000/E digital correlation. The scattering intensity was measured five times and each measurement itself took 200 s.

### 3.3. Dynamical Differential Scanning Measurement

**Figure 3:**
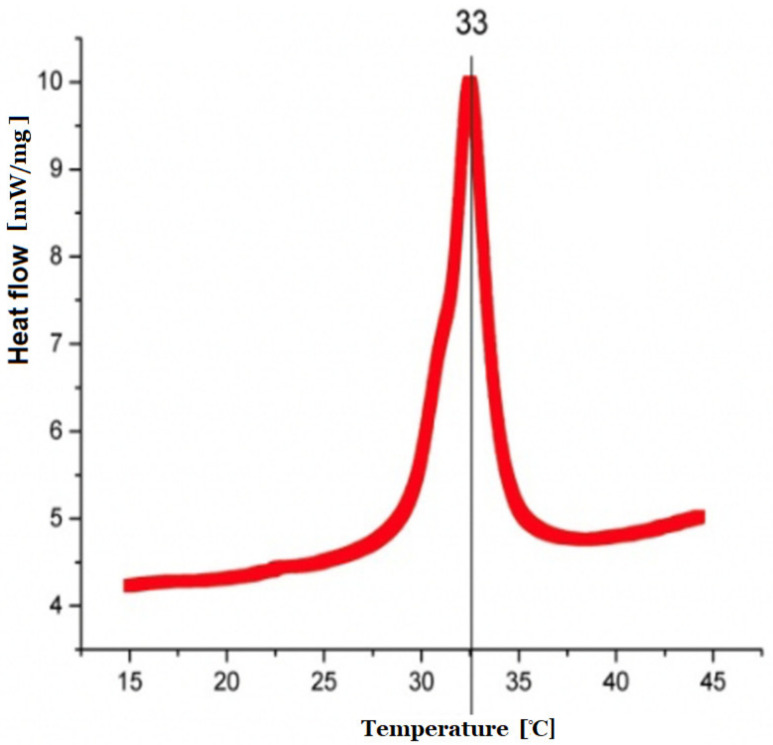
DSC measurement with characteristic increase in heat flow representing phase transition.

Dynamical differential scanning (DLS) calorimetry confirmed that 33 °C was the temperature of phase transition (Figure 3). This temperature represented the LCTS temperature of obtained polymeric particles, which is important during loading and releasing substances of interest. 

### 3.4. Examination of Cargo Loading and Release

We were able to verify and compare the efficiency of loading and release of two model substances that were purposely set by different sizes (BSA and doxorubicin).

**Figure 4:**
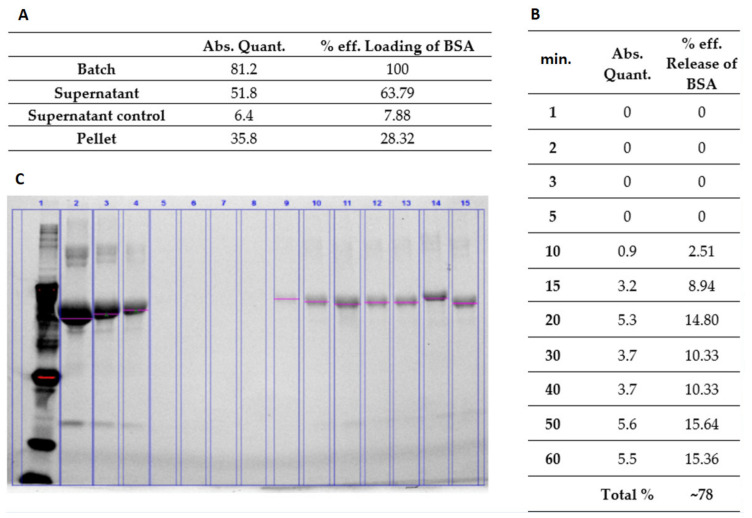
(**A**) Quantification of cargo loading. (**B**) Quantification of cargo release at the appropriate time points. (**C**) Electrophoresis of BSA model-protein in prestained gradient gel. Molecular weight markers are in lane 1 and standard concentrations of 100, 80, and 50 µg/mL of BSA in lanes 2–4. Lanes 5–15 represent BSA released in time periods at 1, 2, 3, 5, 10, 15, 20, 30, 40, 50, and 60 min, respectively.

Both, BSA and DOXO cargos were loaded into synthetized nanoparticles. After loading the BSA, we detected that 63% of the applied cargo protein was present in supernatant while 28% of BSA was detected in the cell pellets. Additionally, using SDS-PAGE electrophoresis we showed the dynamics of cargo release. During the first five min, there was nondetectable BSA cargo in the supernatant. Small amounts of the examined protein were detected at 10 min after reaching LCTS temperature. After 60 min, we detected a high amount of the released tested protein (Figure 4). This may prove a gradual release of cargo (78%) in a rather short time (Figure 4C).

**Figure 5:**
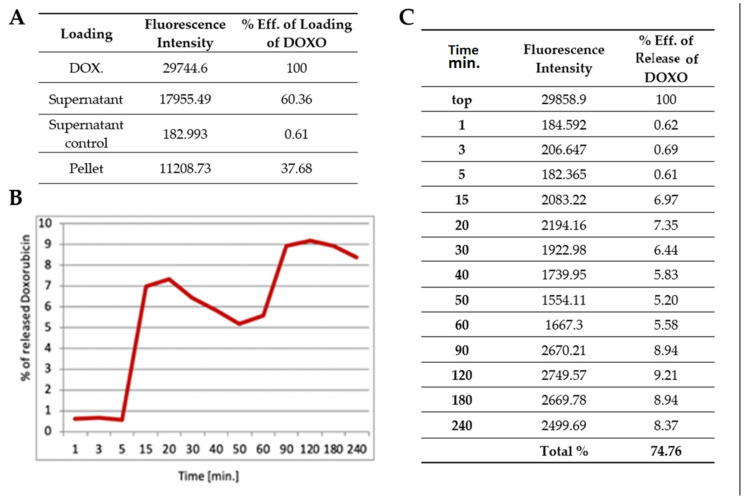
(**A**) Quantification of cargo loading. (**B**) Release kinetics for doxorubicin in first 240 min. (**C**) Quantification of cargo release at the appropriate time points.

Doxorubicin (DOXO), a fluorescent protein, was also loaded into nanoparticles. DOXO particles are relatively larger than BSA. We confirmed that 37% of nanoparticles were with cell pellets loaded with the drug, while 60% of DOXO was detected in the supernatant. The releasing profile showed that 240 min after LCT transition the cargo was still excreted from pNiPAMs (Figure 5B). In summary, approximately 74% of loaded cargo was released during 240 min, as shown in Figure 5C.

In summary, the presented results of the test substances (BSA and DOXO) loaded into nanoparticles were shown to be more stable for the larger molecule—doxorubicin, while the release profile was similar for both substances used in the study. Thus, both large (DOXO, Mw. 543.519 Da) and small (BSA, Mw. 66 430 Da) cargoes were successfully proved to be loaded and released.

### 3.5. BCB Concentration Standardization

**Figure 6:**
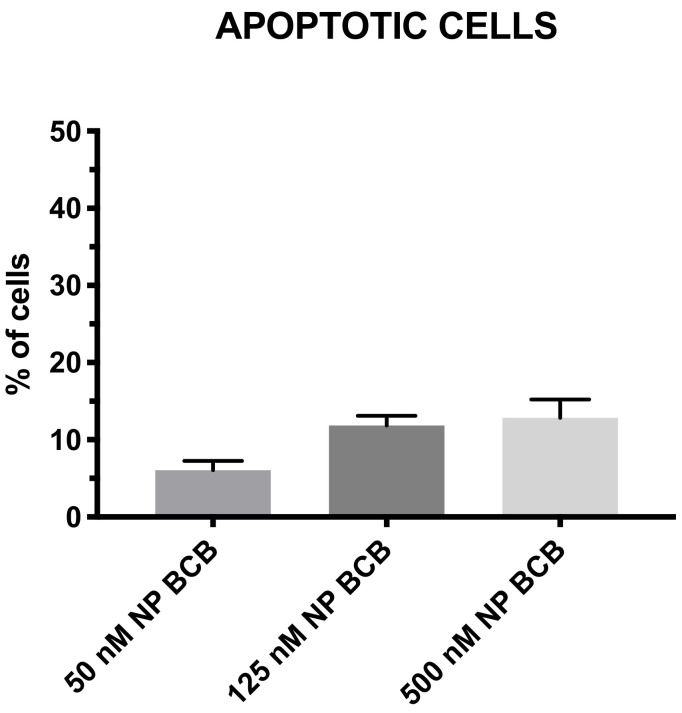
Standardization of BCB-nanoparticles’ concentration effect. BCB-nanoparticles were loaded with different concentration of BCB, *n* = 4. There were no significant differences noted of nanoparticles’ BCB concentrations on spontaneous cell apoptosis after treatment with N-BCB. Abbreviation: NP BCB (nanoparticles bax channel blocker).

Different concentrations of BCB (50−500 nM) were applied in order to verify the biological effect of drug dosing to SkMCs (Figure 6). Each of the BCB tested concentration did not induce significant apoptosis in SkMCs. However, cells treated with 50nM BCB exhibited the smallest level of apoptosis in subjected cells, while the 500 nM demonstrated the highest level (although statistically nonsignificant) Even so, it is worth noting that neither of the proposed BCB concentrations was toxic to tested SkMCs affecting their viability (Figure 6).

### 3.6. Apoptosis Detection/ Visualisation

**Figure 7:**
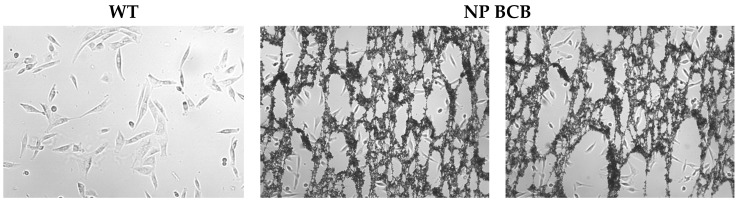
Photographs of SkM cells either of wildtype (WT) or incubated with nanoparticles loaded with BCB (NP-BCB).

After 24 h incubation of SkMC with the nanoparticles loaded with BCB, we visualized the polymeric structures surrounding and attached to the SkM cells, as shown in Figure 7.

**Figure 8:**
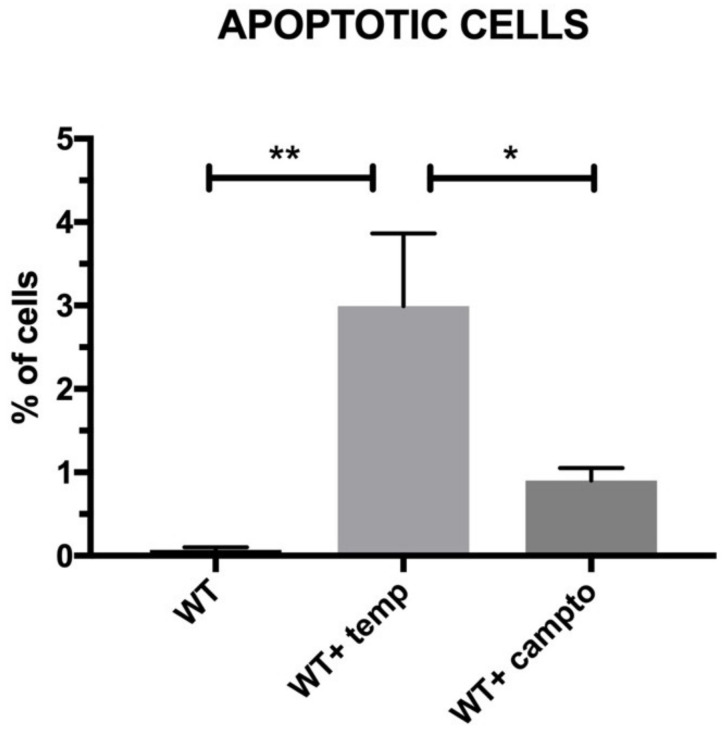
Induction of necrosis/apoptosis by two different treatments of wild type (WT) SkM cells, *n* = 3. Some samples were treated by temperature 90 °C/10 min and the other with camptothecin. Statistical significance has been shown: *p* < 0.05 (*), *p* < 0.01 (**), *p* < 0.001 (***). Results were performed using the one-way ANOVA test.

As an inductor of necrosis/apoptosis, we used high temperature (incubation of cells for 10 min at 90 °C) while camptothecin was used to induce apoptosis. In both examined samples, we detected significantly higher amounts of apoptotic cells in comparison to nontreated (wildtype) SkMCs (Figure 8).

**Figure 9:**
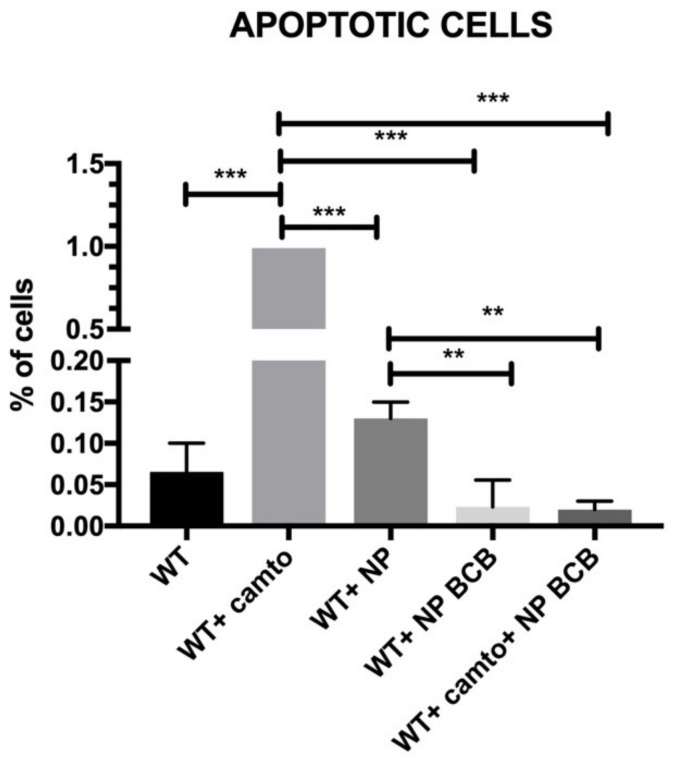
Apoptosis of examined SkMC populations, *n*=3. Abbreviations: WT—wildtype cells; WT campto—wildtype cells treated with camptothecin; NP—nanoparticles; NP BCB (bax channel blocker)—nanoparticles loaded with BCB. Statistical significance legend: *p* < 0.05 (*), *p* < 0.01 (**), *p* < 0.001 (***). Results were performed using a one-way ANOVA.

Next, we examined the level of apoptosis in SkM cells that were incubated with NP either unloaded or loaded with BCB, as shown in Figure 9. The induction of apoptosis was carried out using camptothecin. Wild type cells treated with apoptosis inductor exhibited significant amounts of FITC- (Annexin V) positive cells. Application of empty nanoparticles slightly diminished the apoptosis. The population of cells incubated with NP loaded with BCB critically diminished the apoptosis in a statistically significant way. Moreover, the lowest FITC-positive signals were detected in SkMCs treated with camptothecin and simultaneously incubated with NP loaded with BCB. We note that populations of myoblasts cultured with NP loaded with BCB either treated or nontreated with camptothecin exhibited the lowest percentages of apoptotic cells among the cell populations under study.

**Figure 10:**
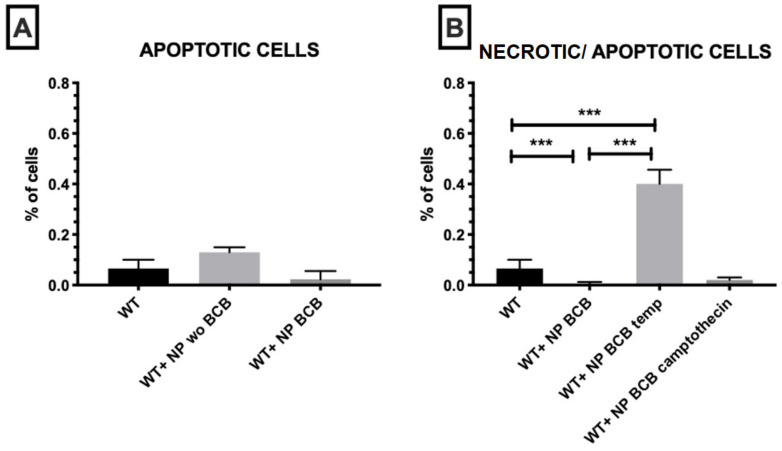
Necrosis/apoptosis observed in examined myoblast cell populations influenced by various types of inductors. (**A**) apoptosis of SkM cells incubated with nanoparticles loaded or unloaded with BCB in comparison to wildtype cells, *n* = 3. (**B**) induction of necrosis and programmed cell death by SkM incubation in high temperature or treated with camptothecin, *n* = 3. Respectively, statistical significance legend: *p* < 0.05 (*), *p* < 0.01 (**), *p* < 0.001 (***). Results were obtained using the one-way ANOVA test. Abbreviations: WT—wild type cells; WT campto—wild type cells treated with camptothecin; NP—nanoparticles; NP BCB (bax channel blocker)—nanoparticles loaded with BCB.

NP without BCB exhibited less effectively the percentage of revealed apoptotic cells in comparison to NP loaded with BCB, although this was not statistically significant (Figure 10A). It is worth noting that in comparison to wildtype SkM, cells incubated with NP (loaded with BCB) demonstrated lower amounts of Annexin V-positive cells (Figure 10A). Still the highest percentage of necrotic/apoptotic cells was detected in the population of cells subjected to high temperature even after application of NP BCB (Figure 10B).

**Figure 11:**
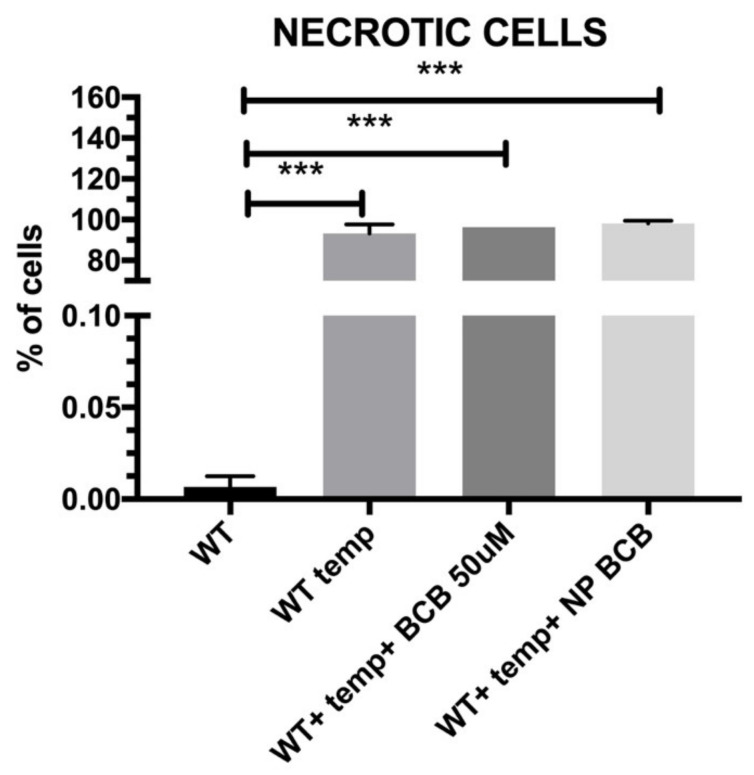
Examination of necrosis in studied populations of WT-SkMCs subjected to high temperature, *n* = 3; WT cells alone; WT cells incubated at 90 °C/10min; SkMCs cells incubated with nanoparticles loaded with BCB and subjected to 90 °C/10 min. Statistical significance legend: *p* < 0.05 (*), *p* < 0.01 (**), *p* < 0.001 (***). Results were obtained using a one-way ANOVA test. Abbreviations: SkMCs—skeletal muscle stem/progenitor cells, WT—wildtype SkMCs, BCB—bax channel blocker, NP BCB–nanoparticles loaded with bax channel blocker.

We next confirmed the nature of death in (nonviable cells) SkMC suspensions subjected to high temperature, which exhibited the high amount of 7-AAD positive cells, as shown in Figure 11. Application of nanoparticles loaded with BCB or subjected to BCB alone did not apparently influence the level of necrotic cells. This may prove that either BCB or NP-BCB did not influence a pathway directed to the cell death in result of necrosis.

## 4. Discussion

So far, one of the most famous polymers due to its biodegradability and good stability in water without any serious side effects has been PLGA [18]. These types of nanoparticles have been extensively studied in cancer research [19]. However, the Poly(N-isopropylacrylamide) (pNiPAM) microgel is perhaps the most well-known nanomaterial. Combining the strengths of hydrogel and nanoparticles, with unique stimuli responsivity in respect to temperature, pNiPAM microgels were found to perform numerous biomedical applications, such as drug delivery [20]. The most important property of pNiPAM microgel is its thermosensitivity [21]. At room temperature, pNiPAM is hydrophilic, and the microgel particles are highly swollen. pNiPAm, which was largely studied in terms of its properties, has a low cost and low risk methods of synthesis and functionalization, so it appears to be a good biocompatible material.

Above a critical temperature, the pNiPAM polymer becomes hydrophobic, so the particles shrink sharply and a cargo is efficiently released. Therefore, pNiPAM microgels are good carriers for protein/peptide origin drugs. As a drug carrier, microgels may also protect the drugs from enzymatic degradation. In this study, a common protein (BSA) as well as a commonly used drug (doxorubicin) [13] were used to test the loading and cargo release capability of pNiPAM-HEMA-AA-oligoLA nanoparticles. Bovine serum albumin is a protein with molecular weight around 69 g/mol while doxorubicin is almost ten times larger with a molecular weight of 579.98 g/mol. The percentage of loading was estimated at 28.32% and 37.68%, respectively, for BSA and DOX. Higher loading in the DOX model may be a result of a small mesh size in nanoparticle structure, which enabled smaller molecules (BSA) to diffuse and leak from the nanospheres while molecules of DOX (which are roughly ten times greater than BSA) were effectively trapped inside the nanoparticle structure. Release of a cargo in both models was consistent, ~78% within one hour for BSA and ~75% within four hours for DOX suggesting shrinking/collapse capability of synthesized nanoparticles, which is critical to achieve a stable drug delivery. Control over the permeability of a drug diffusing from the inside trap to the outside of microgel is a key element for their application as a controlled drug delivery system [22].

Studies on pNiPAM have also focused on cancer treatment, however in the literature, there is also evidence that this system is also suitable for drug delivery in ischemic conditions [23]. Stability and viability in a hostile environment is a great advantage in cell transplantation, e.g., to the postinfarcted heart where hypoxia is a common phenomenon. Nanoparticles can be used in the near future to deliver in situ the drug of interest, e.g., in an ischemic heart. In order to enhance the cellular graft survival versus apoptosis, the cells could be loaded with nanoparticles containing variety of biological substrates.

Apoptosis is a principal mechanism of cell death, which is involved in regulation of tissue homeostasis in multitissue organisms. Several intracellular signaling pathways have been identified for apoptosis, including a mitochondrial one and the death receptor external pathway. Bcl-2 family is the most extensively studied group of proteins and it is composed of pro- (e.g., BAX and Bad) and antiapoptotic (e.g., Bcl-2 and Bcl-XL) members. It is clear that BAX has a central function in the regulation of the mitochondrial apoptotic pathway. BAX is and responds to a diverse range of different stimuli. Blockade of BAX channel activity may inhibit mitochondria-mediated apoptosis in cells in vitro or putatively in vivo. Specific Bax channel inhibitors (BCIs) were developed [24,25]. Some of the molecule inhibitors of the Bcl-2 family have been described, among them inhibitors of cytochrome c release (BCB). Regarding the results from our experiments on SkM cells’ apoptosis and/or necrosis, the pNiPAM-HEMA-AA-oligoLA nanoparticles were successfully used as a potential drug delivery system preventing apoptosis (but not necrosis). Such a system may be used both towards helmeted cardiomyocytes after MI shock as well as to decrease apoptosis in implanted to myocardium stem cell grafts. Using nanoparticles loaded with BCB, we could well promote cell survival in the hostile environment of a postinfarction scar. Most importantly, delivery of the nanoparticles with/without antiapoptotic agents (BCB) was successful. Finally, it was shown that applied BCB molecule inhibited apoptosis induced by camptothecin, however, it did not affect necrosis induced by high temperature. This, radical procedure, however, (90 °C heating) would not take place in the live myocardium.

## 5. Conclusions

The temperature of phase transition represents the LCTS temperature of obtained polymeric particles at 33 °C. BSA and DOXO loaded into nanoparticles showed better stability for the larger molecule (doxorubicin), however, both large (DOXO, Mw. 543.519 Da) and small (BSA, Mw. 66 430 Da) cargoes could be successfully loaded and released by using the pNiPAM system.

SkMCs cultured with pNiPAM-loaded BCB (Bax channel blocker) either treated or nontreated with camptothecin (apoptosis inducer) exhibited the lowest percentages of apoptotic cells among the cell populations under study. We claim that apoptosis suppression performed in a controlled way may limit the loss of both cardiac myoblasts and implemented SkMCs for cellular intervention in a failing heart.

## Data Availability

The data used to support the findings of this study are available from the corresponding author upon request.
